# Computational Analysis of High Risk Missense Variant in Human UTY Gene: A Candidate Gene of AZFa Sub-region

**Published:** 2017

**Authors:** Mili Nailwal, Jenabhai Bhathibhai Chauhan

**Affiliations:** - P.G. Department of Genetics, Ashok & Rita Patel Institute of Integrated Study & Research in Biotechnology and Allied Sciences (ARIBAS), Gujarat, India

**Keywords:** AZFa, Computational analysis, Male infertility, Missense, nsSNP, UTY gene

## Abstract

**Background:**

The human Ubiquitously transcribed tetratricopeptide repeat gene, Y-linked (UTY) gene encodes histone demethylase involved in protein-protein interactions. UTY protein evidence at protein level predicted intracellular and secreted protein. UTY is also involved in spermatogenesis process.

**Methods:**

The high-risk non-synonymous single nucleotide polymorphism in the coding region of the UTY gene was screened by SNP database and identified missense variants were subjected to computational analysis to understand the effect on protein function, stability and structure by SIFT, PolyPhen 2, PANTHER, PROVEAN, I-Mutant 2, iPTREE-STAB, ConSurf, ModPred, SPARKS-X, QMEAN, PROCHECK, project HOPE and STRING.

**Results:**

A total of 151 nsSNPs variants were retrieved in UTY gene out of which one missense variant (E18D) was predicted to be damaging or deleterious using SIFT, PolyPhen 2, PANTHER and PROVEAN. Additionally, E18D variant showed less stability, high conservation and having role in post translation modification using i-Mutant 2 and iPTREE-STAB, ConSurf and ModPred, respectively. The predicted 3D model of UTY using SPARKS-X with z-score of 15.16 was generated and validated via QMEAN (Z-score of 0.472) and PROCHECK which plots Ramachandran plot (85.3% residues in most favored regions, 12.3% in additionally allowed regions, 2.0% in generously allowed regions and 4.0% were in disallowed regions) and it indicates a good quality model. STRING showed that UTY interacts with ten different proteins.

**Conclusion:**

This study revealed that SNP data available on database was deduced to find out the most damaging nsSNPs *i.e*. rs3212293 (E18D). Therefore, it provides useful information about functional SNPs for future prospects concerning infertility in men.

## Introduction

Infertility is a major public health problem in which approximately 15% of couples are not able to conceive an offspring (1) and 30% to 50% of infertile cases are related to only male factors (2). Genetic factor is considered as one of the most important etiology of infertility in men (3). Azoospermia factor (AZF) region on the Y chromosome deletion leads to severe spermatogenetic failure like phenotypic or sperm abnormality (4). AZF region in the long arm of Y chromosome (Yq11) comprises three main sub-regions namely AZFa, AZFb and AZFc (5) where essential genes are located for spermatogenesis (6). Among all the three sub-regions, AZFa is the smallest with molecular length of approximately 800 *Kb* (7). AZFa sub-region contains three main candidate genes *i.e*. Ubiquitin-specific protease 9 (USP9Y), Dead box on the Y (DBY) and ubiquitously transcribed tetratricopeptide repeat gene, Y-linked (UTY).

It has been reported that human UTY gene is located to band 5C corresponding to AZFa sub-region and this band contains one or more genes that have function in spermatogenesis process and Y-specific growth gene (8). The corresponding UTY gene is located on chromosome Yq11.221 and is also known as Histone demethylase UTY, KDM6AL, KDM6C and UTY1. UTY gene has a total of 20 exons (9) and encodes for enzyme which is rich in tetratricopepetide repeats that may be included in protein-protein interaction (8, 10). The UTY gene is conserved between human and mouse (11). UTY gene is ubiquitously expressed and present in single copy (12).

Single nucleotide polymorphism (SNP) is a variation of single nucleotide which can be found anywhere throughout the genome (13). Mostly SNPs are neutral but some SNPs could predispose human to disease or affect drug response (14). Non-synonymous SNPs are found in coding region which can influence resulting protein structure and/or function with either neutral or deleterious effects (15, 16).

It is believed that autosomal and sex chromosomal abnormality accounts for 10–15% of cases in men with infertility (17). Furthermore, recently it has been suggested that genetic causes of infertility in men comprise Y chromosome microdeletion, genetic mutation and single nucleotide polymorphism as well as chromosomal aberration (18, 19). Therefore, detection of molecular changes in the Yq11 region of infertile males is required. Deleterious SNPs for the UTY gene have not been converted to data through computational analysis. Thus, to inspect the potential association among the genetic mutation and phenotypic variation, different algorithms are used to compute the high risk missense mutations in coding regions which may have impact on protein structure and/or function of UTY. Considering the role played by UTY gene in male infertility, the study aimed to narrow down the candidate non-synonymous SNPs (nsSNPs) for the UTY through computational analysis which may influence the protein structure and/or function that may serve an important role in male infertility.

## Methods

Genomic analysis of UTY was conducted using sorting intolerant from tolerant (SIFT), PolyPhen 2, protein variation effect analyzer (PROVEAN) and protein analysis through evolutionary relationships (PANTHER) to retrieve deleterious mutation followed by stability analysis using I-Mutant 2 and iPTREE-STAB. Additionally, conservation analysis prediction (ConSurf), 3D structure prediction, validation of model, structural effect and protein-protein interaction were done for UTY. Details of each tool are described below and highlighted in table 1.

**Table 1.** Computational approaches available as online tools

**Server**	**Input**	**URL**	**Reference**
**SIFT**	rs id’s	http://sift.jcvi.org/	20
**PolyPhen 2.0**	Protein sequence in FASTA format, Position and Substitution	http://genetics.bwh.harvard.edu/pph2	21
**PANTHER**	Protein sequence, substitution and single organism	http://pantherdb.org/tools/csnpScoreForm.jsp	22
**PROVEAN**	Protein sequence and amino acid variations	http://provean.jcvi.org/index.php	23
**I-Mutant 2.0**	Protein Sequence, position and new residue	http://folding.biofold.org/cgi-bin/i-mutant2.0.cgi	24
**iPTREE-STAB**	Residue of deleted, introduced and neighbore mutation type	http://210.60.98.19/IPTREEr/iptree.htm	25
**Consurf**	Protein sequence	http://consurf.tau.ac.il/	26
**ModPred**	Protein sequence	http://montana.informatics.indiana.edu/ModPred/index.html	27
**SPARKS-X**	Protein Sequence	http://sparks-lab.org/yueyang/server/SPARKS-X/	28
**HOPE Project**	Protein Sequence, select a Residue to Mutate and select Mutation	http://www.cmbi.ru.nl/hope/home	29
**QMEAN**	PDB file model	https://swissmodel.expasy.org/qmean/cgi/index.cgi	30
**PROCHECK**	PDB file model	http://www.ebi.ac.uk/thornton-srv/software/PROCHECK/	31
**STRING**	Protein name and organism	http://string-db.org	32

### Dataset:

UTY SNPs (rsIDs) related data and protein sequence were extracted from the National Center for Biotechnology Information (NCBI- http://www.ncbi.nlm.nih.gov) and related information regarding UTY gene and protein was collected from Online Mendelian Inheritance in Man (OMIM- https://www.omim.org/entry/400009).

### Prediction of functional connection of missense mutation:

Four tools were used namely SIFT, PolyPhen 2, PANTHER and PROVEAN to predict the functional context of missense mutation.

SIFT (Sorting intolerant from tolerant) tool predicts the single amino acid change impact on protein function that would be either tolerated or damaging. The SIFT score will be below or equal to 0.05 when amino acid substitution is predicted damaging and if the score is above 0.05, amino acid substitution is predicted to be tolerating (20). PolyPhen 2 (Polymorphism phenotyping version 2) predicted whether the amino acid substitution is probably damaging, possibly damaging or benign with a score ranging from 0.0 (benign) to 1.0 (damaging).

PANTHER cSNP (Protein analysis through evolutionary relationship- coding SNP) predicts functional effect of amino acid substitution on protein by calculating subPSEC (Substitution position-specific evolutionary conservation) score on the basis of alignment of evolutionary related proteins (33).

PROVEAN (Protein variation effect analyzer) predicts the effect of any type of protein sequence variation consisting of amino acid substitution and in-frame insertion and deletion change on proteins biological function. It analyzes the nsSNPs as deleterious or neutral when the score is below or above the threshold, respectively (23).

### Prediction of stability change upon missense mutation:

Generally, mutation leads to structural stability changes which affect the function of protein. Therefore, the stability check was carried out using I-Mutant 2 and iPTREE-STAB. I-Mutant 2 server prediction is based on support vector machine (SVM) for protein stability change of the folded protein in relation with single point mutation. It predicts direction (Δ ΔG sign) as well as Δ ΔG associated values of the protein stability changes upon single site mutations. iPTREE-STAB server is based on decision tree where it predicts the stability change (Δ ΔG values) due to single site mutation. Thus, it predicts whether the outcome of the amino acid substitution is stabilizing or destabilizing (34).

### Phylogenetic conservational analysis of UTY:

Conservation prediction of UTY amino acids was analyzed using ConSurf tool. It uses high-throughput characterization of functional regions of the protein. The degree of conservation of amino acid is calculated based on conservational score in the scale of 1–9 where 1–3 scores are variable, 4–6 scores indicate average conservation and 7–9 scores indicate high conservation (26).

### Prediction of post translational modification sites for UTY:

In UTY protein, post translational modification (PTM) sites were sequence based, predicted using ModPred. It accommodated 34 ensembles of logistic regression models that were trained independently on a united set of 126,036 non-redundant experimentally verified sites for 23 distinct polymorphism, retrieved from pubic databases and an ad-hoc literature investigation (27).

### Prediction of 3D structure of UTY:

SPARKS-X is a single method fold recognition technique which gives 3D structure. This tool was improved by changing the alignment scoring function as well as adding the SPINE-X techniques which upgraded prediction of secondary structure, backbone torsion angle and solvent accessible surface area (28).

### Model validation for UTY:

The predicted model of UTY from SPARKS-X was validated using qualitative model energy analysis (QMEAN) and PROCHECK. QMEAN z-score was another tool used for quality assessment where it analyzed the degree of nativeness of the predicted 3D structure of protein. The QMEAN score imitated the predicted global model reliability ranging between 0–1 (35). PROCHECK provides a detailed analysis on the stereochemistry quality of the 3D protein structure. Ramachandran plot with Phi/Psi was provided by the PROCHECK to validate the backbone structure of protein.

### Prediction of structural effect of point mutation on UTY:

Project HOPE was used to know the structural effect where it was used for molecular dynamics simulation to analyze the single point mutation. First BLAST was performed against PDB and built a homology model and retrieved tertiary structure information through WHATIF followed by the access to UniProt database features. Furthermore, the protein features were predicted using Distributed Annotation System (DAS) server (29, 36).

### Prediction of protein-protein interactions:

Search Tool for the Retrieval of Interacting Genes/Proteins (STRING) database provides a critical assessment and integration of protein-protein interactions whether it is direct (physical) or indirect (functional) association (37).

## Results

### SNP datasets:

The polymorphism data for UTY gene investigated in present work was retrieved from NCBI dbSNP^1^ database and contained 151 missense, 4 non-sense, 2 splice site at 3′ end, 73 UTR at 3′ end, 1 splice site at 5′ end, 5 UTR at 5′ end, 84 coding synonymous, 6 frame shift, 2302 introns and 4 stop gained SNPs. Only non-synonymous coding SNPs of UTY were selected for this investigation.

### Prediction of functional nsSNPs in UTY:

The UTY single nucleotide variants were administrated to computational analysis through variety of tools.

According to SIFT, a total of 5 out of 151 nsSNPs of UTY gene from dbSNP analysis were predicted to be tolerated or deleterious (Table 2). SIFT classified 4 nsSNPs damaging (E18D, R1015Q, T261P and S824F) where tolerance index score was identified to be 0 (<0.05) and 1 nsSNP (E34G) tolerated with tolerance index score of 0.19. Only 1 missense variant was predicted to be probably damaging (E18D) and the rest 4 missense variants were classified to be benign through PolyPhen 2 output (Table 3).

**Table 2.** nsSNP analysis by SIFT

**Sr. No.**	**SNP**	**Amino acid change**	**Amino acid**	**Using homologues in the protein alignment**	

				**Prediction**	**Score**
**1**	rs9341273	E34G	E	TOLERATED	1
			G	TOLERATED	0.19
**2**	rs3212293	E18D	E	TOLERATED	1
			D	DAMAGING	0
**3**	rs9341281	R1015Q	R	TOLERATED	1
			Q	DAMAGING	0
**4**	rs75596360	T261P	T	TOLERATED	1
			P	DAMAGING	0
**5**	rs112993031	S824F	S	TOLERATED	0.33
			F	DAMAGING	0

**Table 3.** nsSNP analysis by PolyPhen 2

**dbSNP ID**	**Amino acid substitution**	**HumDiv**		**HumVar**	

		**Prediction**	**Score**	**Prediction**	**Score**
**rs61730117**	E34G	Benign	0.002	Benign	0.002
**rs3212293**	E18D	Probably Damaging	0.979	Probably Damaging	0.983
**rs9341281**	R1015Q	Benign	0.006	Benign	0.000
**rs75596360**	T261P	Benign	0.026	Benign	0.027
**rs112993031**	S824F	Benign	0.190	Benign	0.042

To increase the accuracy of computational techniques to compute utmost deleterious SNPs, different computational methods were used. Therefore, PANTHER and PROVEAN were used for further analysis. Out of 5 variants, one (E18D) was predicted to be damaging with SIFT and PolyPhen 2. PANTHER was performed to verify validation of results obtained from two tools and E18D was portrayed damaging with SIFT, PolyPhen 2 and now with PANTHER too (Table 4). E18D was selected for further confirmation through PROVEAN where the output came to be deleterious (Table 4).

**Table 4.** nsSNP analysis by PANTHER and PROVEAN

**Variant**	**PROVEAN**		**PANTHER**

	**PROVEAN Score**	**Prediction (cutoff= −2.5)**	
E18D	−2.536	Deleterious	Possibly damaging

Protein stability of UTY variant upon point mutation was found using I-Mutant 2.0 and iPTREE-STAB. E18D was subjected for stability prediction and showed decreased stability by both the tools (Table 5).

**Table 5.** Stability analysis of E18D variant

**dbSNP ID**	**Amino acid substitution**	**I-Mutant 2.0**			**iPTREE-STAB**	

		**Stability**	**RI**	**DDG**	**The discriminated direction of thermal stability change**	**The predicted value of thermal stability change (Kcal/mol)**
rs61730117	E18D	Decrease	6	−0.57	Decrease	−0.1221

### Conservation profiling of UTY:

Amino acids are highly conserved when located in biologically active sites. If there is substitution of these amino acids, it leads to complete loss of biological activities (38). For this purpose, ConSurf server was used to predict the degree of evolutionary conservation in the protein UTY at each amino acid position. Although there is complete analysis of UTY protein, only those amino acids have been focused which were selected as high risk nsSNP. The conservational analysis revealed that E18D is highly conserved with a score of 8. Also, E18D is a conserved amino acid, so its functional role is very critical.

### Post translational modification sites on UTY:

To analyze the effect of nsSNPs on post translational modification process of human UTY protein, Mod Pred tool was used. ModPred predicted sites for proteolytic cleavage, carboxylation and ADP-ribosylation at E18 (Table 6).

**Table 6.** ModPred analysis of E18D variant

**Residue**	**Modification**	**Score**	**Confidence**
**E18D**	Proteolytic cleavage	0.51	Low
	Carboxylation	0.77	Medium
	ADP-ribosylation	0.63	Low

### 3D modelling and biophysical validation of UTY:

**Figure 1.**
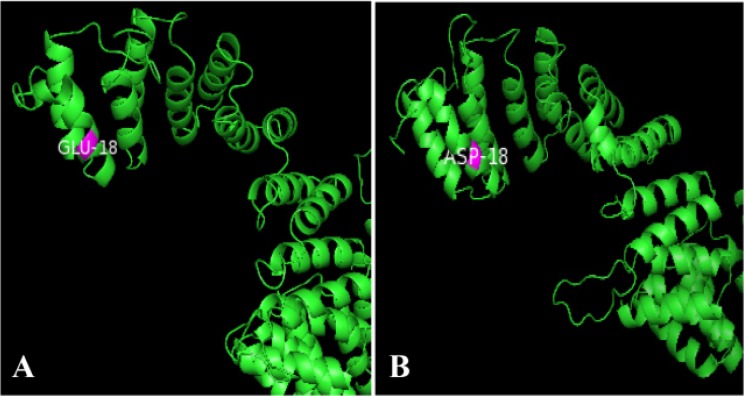
A: Wild-type model showing glutamic acid at position 18 (GLU18) of UTY protein constructed using SPARKS-X and visualized by PyMol. B: Mutant model showing aspartic acid at position 18 (ASP18) of UTY protein constructed using SPARKS-X and visualized by PyMol

SPARKS-X modeled the 3D structure of UTY where 10 full length models were generated. The quality of full length models were predicted by z-score (Z-score >6). Using SPARKS-X, native model as well as mutated model with the most deleterious variant (E18D) was generated (Figure 1).

QMEAN tool was used where z-score gives prediction about the absolute quality of the model (Table 7). The quality was estimated by comparing other similar reference structures (39). The total QMEAN score was 0.472 along with its z-score −3.22 which comes under calculated model reliability value ranging between 0–1. According to SPARKS-X, z-score should be greater than 6 and the predicted model z-score was 15.16. This indicated that the predicted model was of good type.

**Table 7.** QMEAN score of E18D variant

**Parameters**	**Score**
**C_beta interaction energy**	−88.52 (Z-score: −1.71)
**All-atom pairwise energy**	−14526.09 (Z-score: −1.93)
**Solvation energy**	−11.40 (Z-score: −2.67)
**Torsion angle energy**	−104.71 (Z-score: −3.11)
**Secondary structure agreement**	77.1% (Z-score: −0.33)
**Solvent accessibility agreement**	67.8% (Z-score: −2.43)
**Total QMEAN-score**	0.472 (Z-score: −3.22)

**Figure 2.**
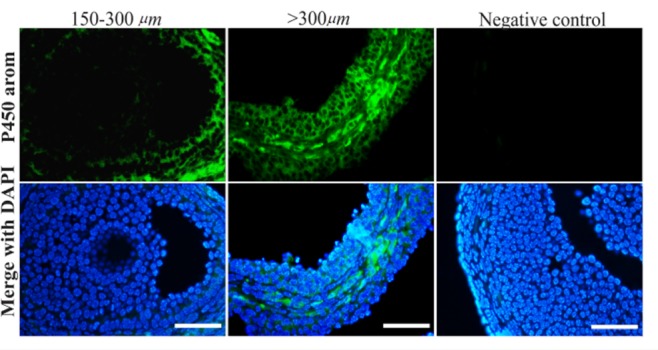
Ramachandran plot of modeled UTY protein

SPARKS-X predicted model was validated using another tool known as PROCHECK where Ramachandran plot was used. Out of all the residues of UTY protein, 1024 (85.3%) were in most favorable regions, 148 (12.3%) were in additionally allowed regions, 24 (2.0%) were in generously allowed regions and 5 (0.4%) were in disallowed regions (Figure 2). Therefore, UTY protein structure can be considered as a relevant model.

**Figure 3.**
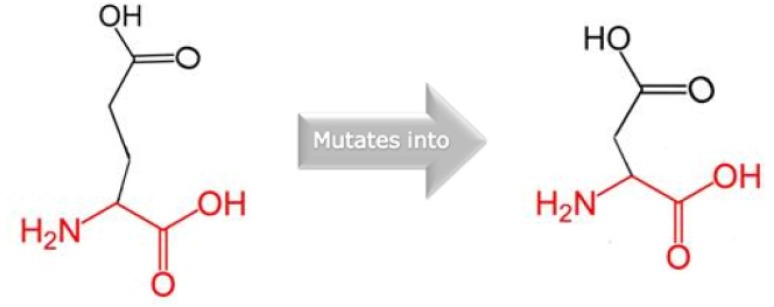
Schematic structures of the original (left) and the mutant (right) amino acid. The backbone, which is the same for each amino acid, is colored red and side chain, unique for each amino acid, is colored black

Project HOPE was used to know the structural changes but no such information was retrieved. Therefore, HOPE used UniProt database and Reprot software was used to predict the mutational analysis. E18D substitution results into a change in glutamic acid residue to an aspartic acid residue at position 18 (Figure 3). The mutated residue (Aspartic acid-D) is smaller than the wild type residue (Glutamic acid-E) which may lead to loss of interaction.

### Analysis of protein-protein interaction:

**Figure 4.**
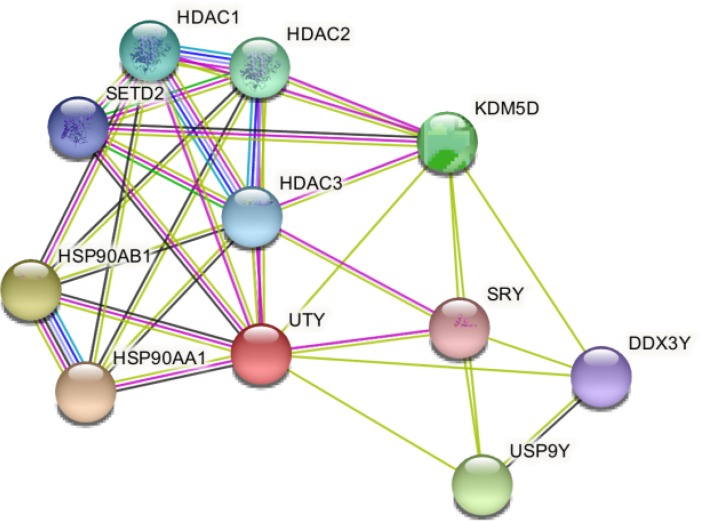
Protein–protein interaction network of UTY using STRING server

STRING prediction indicated that UTY interacts with heat shock protein 90 **kDa** alpha, class A member 1 (HSP90AA1); heat shock protein 90 **kDa** alpha, class B member 1 (HSP90AB1); ubiquitin specific peptidase 9, Y-linked (USP9Y); lysine (K)-specific demethylase 5D (KDM5D); histone deacetylase 2 (HDAC2); histone deacetylase 1 (HDAC1); histone deacetylase 3 (HDAC3); SET domain containing 2 (SETD2); DEAD (Asp-Glu-Ala-Asp) box polypeptide 3, Y-linked (DDX3Y) and sex determining region Y (SRY) (Figure 4).

## Discussion

Single nucleotide polymorphism is the important variant as it accounts for large number of inherited diseases. To functionally distinguish neutral and disease associated SNPs is of great importance. Screening and identification of variants which in turn is responsible for particular phenotypes through molecular approaches is likely to be time consuming, tedious and expensive (14, 40). As the number of SNPs reports are increasing in databases, so it is difficult to choose a target SNP for analysis which will impose contribution in disease development. Therefore, computational path can help to limit the number of SNPs for screening of genetic diseases. Earlier, there were many studies related to polymorphism screening by applying computational approach to predict the functional missense mutations associated with gene like ubiquitin-specific protease 9, Y chromosome, USP9Y (41); Deleted in Azoospermia Like, DAZL (42); Superoxide Dismutase 2, SOD2 (43); Phosphatase and tensin homolog, PTEN (44).

The AZFa sub-region comprises three candidate genes- DDX3Y (former name DBY), USP9Y and UTY (4) and mutation in these genes leads to absence of spermatogenic cells. In human UTY gene, more than 150 missense mutations have been reported to date. However, there is no significant amount of polymorphism studies on UTY which is waiting for extensive studies based on population and clinical screening which may affect male infertility. So, this study might be helpful in understanding the effect of missense mutation on protein function of UTY in relation with infertility in men.

The results obtained in the present study revealed that utilization of various algorithms serves as a dynamic tool to estimate or identify candidate functional nsSNPs. According to Thusberg and Vihinen (2009) and Hicks et al. (2011), SIFT and PolyPhen 2 have been reported as better performing tools to identify most deleterious nsSNPs (45, 46). I-Mutant and iPTREE-STAB were used to check the stability change after single amino acid polymorphism. According to established computational studies, this study included SIFT, PolyPhen 2, PANTHER, PROVEAN, I-Mutant 2 and iPTREE-STAB for the screening and identification of functional mutations in UTY gene. By analyzing all the missense mutations through these tools, one nsSNP with position E18D was predicted to be high risk nsSNP.

The ConSurf tool output indicated that the nsSNP at position E18 was predicted in highly conserved region and may have promising role in UTY protein function. In addition, the E18 residue showed PTM sites for proteolytic cleavage, carboxylation and ADP-ribosylation. Therefore, amino acid polymorphism at E18 position is likely to have impact on post translational modification process of human UTY protein.

For UTY protein, 3 dimensional models were generated using SPARKS-X to visualize deviation between the wild type and mutant type protein models. According to HOPE in E18D, the wild type protein framework was disturbed due to shift of glutamic acid to aspartic acid upon missense mutation which may cause loss of interaction.

Validation of SPARKS-X generated model using QMEAN and PROCHECK servers indicated that model of UTY is good for further experiment as well as for better understanding of biological activity of UTY protein. Project HOPE was used to understand structural changes upon amino acid polymorphism. The wild and mutant amino acids differ sometimes due to specific properties which can disturb the structural and/or functional features of the native protein (47). Similarly, in missense mutation in the UTY protein at amino acid position 18 where Glu is converted to Asp, the mutant residue is smaller and loss of interaction can occur because of the smallness. Analysis of protein-protein interaction is one of the best ways to find out the organization of proteomes related to functional network (48). The use of functional network view for a particular genome is carried out to refine the statistical potential for human molecular genetics (49). STRING was used for protein-protein interaction analysis; reported interaction of UTY protein with different proteins may have an impact on many other pathways involved in the disease.

## Conclusion

The present study symbolized the first detailed analysis where sequence and structure based algorithms are used to identify functional nsSNPs in UTY gene. Out of 151 nsSNPs, 1 (0.66%) was predicted to be deleterious by SIFT and PolyPhen 2. Further analyses support that E18D variant is predicted to cause change in stability and functional interaction performance of UTY protein. This computational analysis of UTY will assist to understand and design future experimental research.

## Footnotes

1- DataBase of Single Nucleotide Polymorphism
